# Biomechanical responses of peripheral nerves in human, pig and rat: a comparative study

**DOI:** 10.3389/fbioe.2025.1641386

**Published:** 2025-12-04

**Authors:** Anita Singh, Scott Kozin, Sriram Balasubramanian

**Affiliations:** 1 Bioengineering Department, Temple University, Philadelphia, PA, United States; 2 Shriners Children’s, Philadelphia, PA, United States; 3 School of Biomedical Engineering, Science and Health Systems, Drexel University, Philadelphia, PA, United States

**Keywords:** peripheral nerve, tension, injury, human, pig, rat, biomechanics

## Abstract

Peripheral nerve injury primarily results from trauma and understanding their mechanical responses is critical to both, the prevention, and the management of sustained injuries. This study aimed to determine and compare the biomechanical responses of sural nerve in human infants, and age-equivalent pig and rat animal models. Biomechanical failure tensile testing was performed on freshly harvested sural nerves. Obtained failure load, and calculated failure stress, corresponding strain, and Young’s Modulus (E) were compared among various species. Histological analysis was also performed on tested nerves to determine the extent of vascular and fiber damage. No significant differences in the failure properties of the age-equivalent human and pig sural nerves were observed. However, the failure load and E values were significantly higher in human and pig nerves when compared to rat nerves. Failure stress was significantly higher in humans than in rat nerves. Histological analysis reported non-significant species-specific differences. In summary, rat sural nerves reported significantly weaker biomechanical properties while the observed structural damage was similar in the three studied species. Obtained data offers an understanding of species-specific differences in the nerve biomechanical properties and can guide translational research that aims to advance the area of peripheral nerve injury and repair.

## 1 Introduction

Peripheral nerve injury (PNI) with a reported incidence of 11.2 events per 100,000 population leads to motor and sensory impairments and is a serious health problem conferring life-long effects. PNI primarily results from trauma and understanding the mechanical responses of the peripheral nerve is critical to both, the prevention as well as the management of sustained injuries. Several animal models have been employed to understand PNI responses when subjected to mechanical forces. These studies have illustrated the biomechanical behavior of peripheral nerves under non-physiological loading conditions while reporting the nerve tissue failure load, stress, strain, and modulus of elasticity values (Rydevik et al., 1990; Wall et al., 1992; Fung, 1993; Ma et al., 2013; Sunderland and Bradley, 1961; Zapałowicz and Radek, 2005; Millesi et al., 1995). Some of these studies have also correlated the resulting neurophysiological and histological changes within the tissue at varying mechanical loading conditions. Together, these studies have confirmed that the injury severity is directly related to the extent of subjected mechanical force/displacement (Rydevik et al., 1990; Wall et al., 1992; Sunderland, 1951; Gör and Rydevik, 1973; Kwan et al., 1992; Ochs et al., 2000; Takai et al., 2002). Despite the extensively published work that provides valuable insights into the biomechanics of peripheral nerves when subjected to loading, the biomechanical properties of peripheral nerves remain poorly understood. The reported failure properties, among various animal models and human cadaveric tissue, exhibit a wide range of failure loads from 1 to 630 N, and failure strain of 5%–58% at corresponding failure stress of 0.25–49 MPa (Kawai et al., 1989; Kleinrensink et al., 2000; Marani et al., 1993; Narakas, 1993; Singh et al., 2009; Singh et al., 2006a; Singh et al., 2006b). The modulus of elasticity values also varies widely and is reported to be between 0.1 and 28 MPa. Variations in loading rate, type of peripheral nerve, age, and species most likely contribute to the reported wide range of biomechanical properties of peripheral nerves.

While the effects of loading rate, age and type of peripheral nerve on the biomechanical properties of peripheral nerves are well documented, the species-specific differences remain poorly understood (Ma et al., 2013; Zapałowicz and Radek, 2005; Millesi et al., 1995; Marani et al., 1993; Singh et al., 2009; Singh et al., 2006a; Singh et al., 2006b; Zapałowicz and Radek, 2000; Singh, 2017; Kallakuri et al., 2004; Destandau et al., 1986; Topp and Boyd, 2006). The non-homogeneous nature of peripheral nerves among various species, primarily due to variations in the proportions of the nerve components, is well documented. These compositional differences in the nerve structure, among various species, warrant studies that offer a clear understanding of any differences in the biomechanical responses of nerves in species that offer promising data for translational research. While a few published studies highlight species-specific differences when comparing small animal models such as rats and mice to large animal models including dogs, cats, and swine, no study has compared animal nerve tissue with fresh human nerve tissue (Giannessi et al., 2017; D’Andrea et al., 2021).

Furthermore, most of the reported studies on peripheral nerves use adult animals and cadavers (Kawai et al., 1989; Kleinrensink et al., 2000; Marani et al., 1993; Narakas, 1993; Singh et al., 2009; Singh et al., 2006a; Singh et al., 2006b). While adult peripheral nerve injuries are prevalent, the occurrence of PNI is even more prevalent, and one of the most reported injuries, in the pediatric population with an occurrence of 10%–15% (Birch and Achan, 2000). These injuries, similar to adults, result primarily from trauma. Few studies have reported the effect of age on nerve biomechanics and repair. However, species-specific comparative studies are not available in the peripheral nerves of young age-equivalent animal models and human tissue. Quantifying the variations in the peripheral nerve biomechanical responses among various age-equivalent young experimental animal models and humans is critical to translating the injury outcomes and for developing promising intervention strategies in the pediatric population (Birch and Achan, 2000). This study aimed to fill this critical gap by determining the biomechanical responses of sural nerve in human infants, and age-equivalent pig and rat animal models, and furthermore, comparing their biomechanical responses for a better understanding of species-specific differences. Such understanding can help guide translational research that aim to advance the area of peripheral nerve injury and repair.

## 2 Materials and methods

All procedures used in the study were approved by the Institutional Review Board. Informed consent was obtained from all subjects involved in the study. Animal studies were in strict accordance with the recommendations in the Guide for the Care and Use of Laboratory Animals of the National Institutes of Health. The protocol was approved by the Institutional Committee on the Ethics of Animal Experiments. All experimental steps were performed with no suffering in the animals since this is an *ex vivo* study.

### 2.1 Tissue harvest

Sural nerves harvested intra-operatively from 15 human infants (4–6 months old, n = 15), 10 age-equivalent pigs (3–4 weeks old, n = 20), and 12 age-equivalent rats (10 days old, n = 24) were used in this *ex-vivo* study. In humans, the subject was placed prone, and the sural nerve was isolated between the lateral malleolus and Achilles tendon. Through a series of transverse incisions, the sural nerve was traced to the popliteal fossa. Typically, the sural nerve originated from the peroneal nerve. Occasionally, there was a communicating branch to the tibial nerve. The nerve was freed from the popliteal fossa to the lateral malleolus using sharp dissection and separating the nerve from its connections in the calf. Once the nerve was dissected free, the origin was divided from the peroneal and/or tibial nerve. The nerves were placed in a Telfa sponge, soaked in saline, and placed on ice until testing. In animals, immediately after euthanizing, using blunt dissection techniques, both right and left sural nerves were carefully harvested from their origin in the hip (a few millimeters distal to the greater trochanter) through their distal branching at the lateral malleolus level. The freshly harvested samples were placed on saline-soaked gauze until testing. All testing was performed within 1–2 h post-harvest. For this study, we utilized the anatomically identified sural nerve for each species. This study aimed to highlight species-specific differences that could be related to anatomical variability, including the location of the nerve. Typical lengths of 3–4 cm nerves were harvested from human subjects, and 1–2 cm nerves from animals bilaterally (pigs and rats).

### 2.2 Biomechanical test setup

**FIGURE 1:**
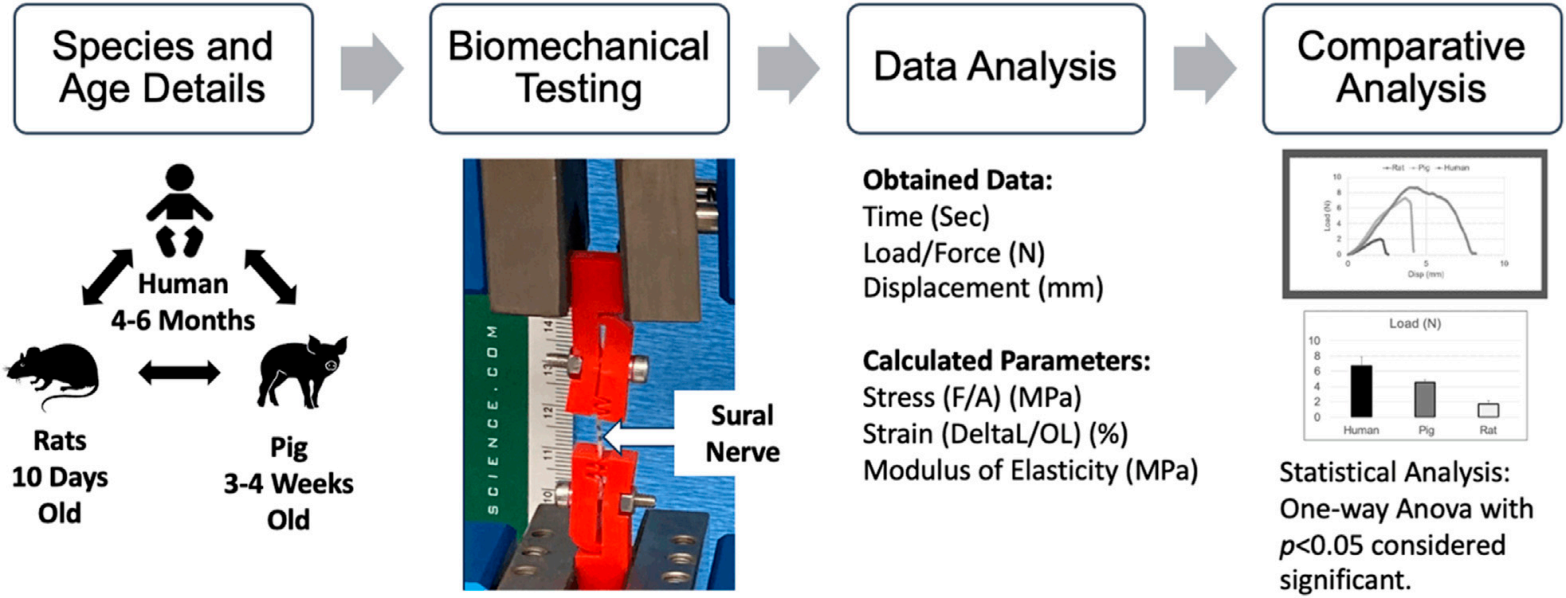
Biomechanical testing details including species and their ages, biomechanical testing set-up, data acquisition, and analysis and statistical analysis. F: Force (N), A: Nerve Area (mm2), DeltaL: Change in Length (mm), OL: Original Length (mm).

An ADMET material testing machine (eXpert 7600, ADMET Inc., Norwood, MA, United States) was used to perform tensile testing of the harvested sural nerves. As shown in Figure 1, a harvested sural nerve was attached to the biomechanical testing setup using customized nerve clamps (detailed in (Singh et al., 2006b)). Briefly, these spring-loaded clamps with serrated jaws help minimize clamping stress while securing the tissue to avoid slippage during tensile testing. The clamps were secured to the mechanical testing machine and a 50 N load cell was used to measure the load subjected to the nerve during tensile testing.

### 2.3 Camera system setup

During testing, a Basler acA640-120uc high-speed video camera (Basler Inc., Exton, PA, United States) was positioned in front of the material testing machine to capture the images of the nerve during testing, which were later analyzed using a customized Matlab code (Orozco et al., 2024). Two markers, using India ink, were placed closer to the clamped ends of the nerve. The camera acquired images at a sampling rate of 120 fps, which were later analyzed to determine the strain on the nerve by tracking the positions of the two markers placed on the nerve just prior to testing.

### 2.4 Testing procedures

Before testing the nerve, a digital microscope (5X; Digital VHX Microscope, Elmwood Park, NJ, United States) was used to obtain images of harvested sural nerves. A 2 mm ruler (Leitz, Ernst-Leitz-Wetzlar GmbH, Germany) was imaged at the same magnification to measure the nerve tissue diameter. The samples were then clamped and stretched uniaxially at a pre-determined displacement rate of 500 mm/min until failure was observed. Failure was confirmed by visual inspection and zeroing of the load-time plot that was acquired in real-time during testing (Figure 1).

### 2.5 Data analysis

The load, displacement, and time data, obtained during tensile failure testing, were utilized for data analysis. Load data was used to calculate nominal stresses (i.e., load/original cross-sectional area of the sample). The strain values were determined from the image analysis of the markers. Details of the marker-based strain analysis have been published previously (Singh et al., 2018). The load-displacement and stress-strain curves were then plotted, and the failure load/force (N), failure stress (MPa), corresponding strain (%), and Modulus of Elasticity (MPa, slope of the linear region of the stress-strain curve) values were determined as described previously (Singh et al., 2006b; Singh et al., 2018).

### 2.6 Histology

Tissue Preparation: Immediately post-testing, the tissue was unclamped and the stretched sections of the nerve tissue were fixed using 4% formalin for 48 h and then paraffin embedded for routine histological sectioning. 7 μm thick longitudinal sections were obtained and stained with hematoxylin–eosin (H&E). Six-eight control (unstretched) samples were also used for histological studies for each of the studied species.

**FIGURE 2:**
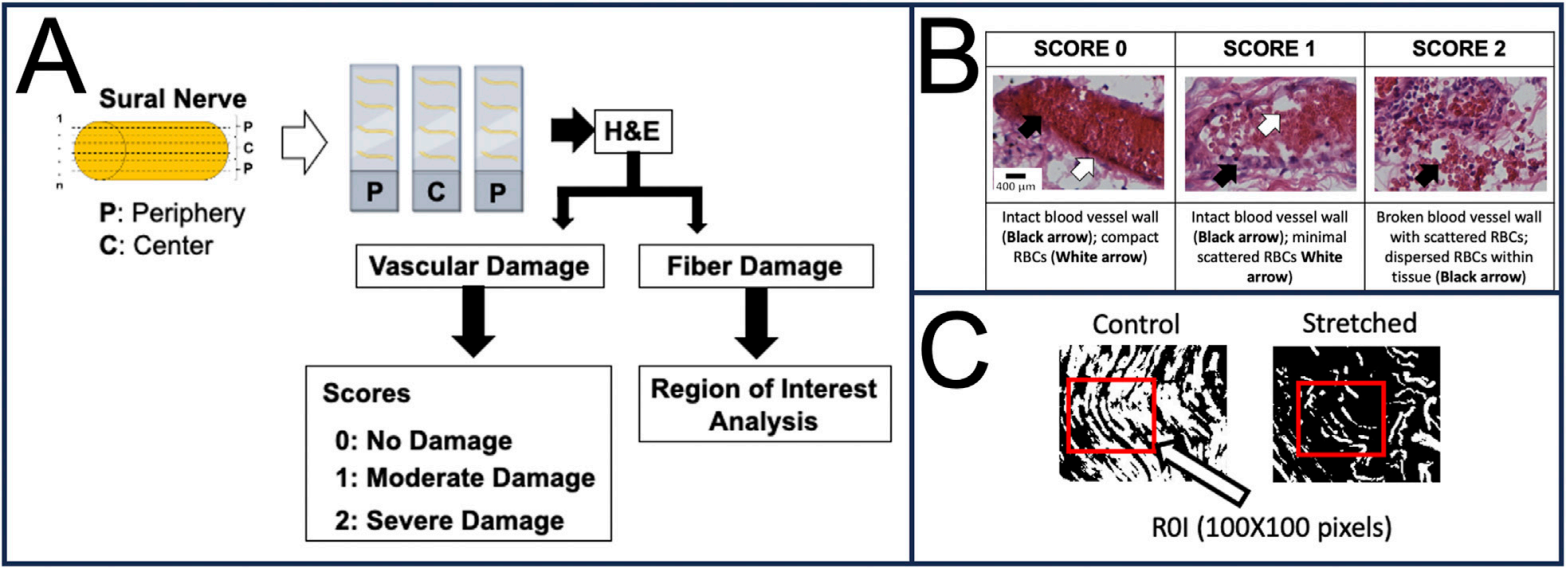
Histological study details. **(A)** Methods details. **(B)** Blood vessel scoring details. **(C)** The extent of fiber damage was quantified using Image J. Region of Interest (ROI) analysis of binarized nerve image (10x). Fixed ROI (100 × 100 Pixels) was placed at a randomly assigned region of the nerve and the amount of fiber (Control: Black, stretched: White) over the selected area was measured. H&E: Hematoxylin and Eosin.

Histological Staining: Every slide had four longitudinal serial sections (Figure 2A). For staining, a single slide was chosen from the two peripheral sides and one from the neural axis also referred to as the center of the nerve. These three stained slides per sample were then used to quantify vascular damage as well as the extent of fiber damage.

Histological Analysis: To quantify the structural damage within the nerve sample, a custom scoring technique was employed. Using the Olympus BX53 motorized microscope, the H&E slides were imaged along the entire nerve length at 10X magnification. Typically, a total of 7–10 regions of 10X magnification were obtained for each nerve section.

Vascular Damage: Each region was carefully observed for the presence of blood vessels (BVs) and scored between 0 and 2 by a blinded observer. A value of 0 indicated none to minimal damage, where the BV wall remained intact and red blood cells (RBCs) remained compact, a value of 1 indicated moderate damage, where the BV walls remained intact with increased spacing and minimal scattering of RBCs, and a value of 2 indicated severe damage, where there were broken BV walls with scattered RBCs and/or dispersed RBCs within the tissue (Figure 2B).

Fiber Damage: To quantify the extent of fiber damage, ImageJ (National Institute of Health, MD, United States) was used to first binarize the nerve images and then create a region of interest (ROI: 100 × 100-pixel square) that was placed over the most damaged region of the nerve section. This was performed for all four sections of the three studied slides per nerve. The obtained values corresponded to the area occupied by the nerve fascicles within this ROI and helped quantify the extent of fiber damage (Figure 2C).

### 2.7 Statistical analysis

Statistical analysis was performed using SPSS software (IBM, Chicago, IL). Values were expressed as Average ±standard error of mean (SEM). Based on the normality of data determined by the Shapiro-Wilk test, parametric tests were performed for statistical analyses. Nerve diameter and all obtained and calculated biomechanical testing parameters (Failure Load, Failure Stress, Strain at Failure Stress, and E) were compared using a one-way ANOVA. Subsequent pairwise comparisons were conducted with a Bonferroni correction. A similar analysis was performed for the quantified structural damage including the score for blood vessel rupture and the extent of nerve fiber damage among the three species, in both the control as well as the tested specimens. For all analyses, *p* < 0.05 was considered significant and *p* < 0.1 was considered moderately significant.

## 3 Results

A total of 15 human, 20 pig, and 24 rat sural nerves were tested for failure under tensile loading in this *ex-vivo* study. Slippage from the clamps was observed in 20%, 25%, and 17% of the tested human, pig, and rat sural nerves, respectively. Slips were confirmed from the camera images, strain response, and load data. A steep decrease in strain and load data were observed during slips. No tissue inside the clamp also confirmed slippage of the tissue from within the clamp. These slipped samples were excluded from the study.

### 3.1 Biomechanical responses

**FIGURE 3:**
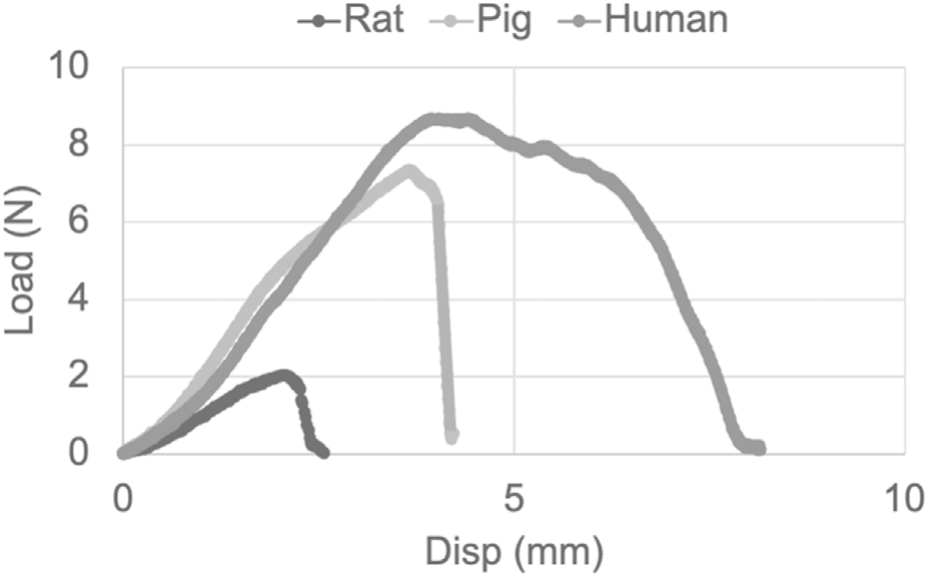
Exemplar load-displacement responses of age-equivalent human, pig, and rat sural nerves when subjected to tensile testing until failure.

Typical load-displacement behavior was reported in the sural nerves from all the species (Figure 3). The reported diameter and failure load, and the calculated failure stress, strain at failure stress, and E were summarized and compared among the three age-equivalent species (Table 1).

**TABLE 1:** Summary of biomechanical tensile properties (Average ± SEM) of human infant (4–6 months), pig (3–4 weeks), and rat (10 days) peripheral nerve. n: Sample size.

Species (Sample size)	Failure load (N)	Failure stress (MPa)	Strain (%) at failure stress	E (MPa)
Human (n = 12)	6.70 ± 1.30	10.45 ± 2.91	36.94 ± 10.7	32.09 ± 6.10
Pig (n = 15)	4.57 ± 0.33	8.64 ± 0.99	41.3 ± 3.44	30.42 ± 3.34
Rat (n = 20)	1.73 ± 0.44	4.54 ± 1.4	47.29 ± 2.65	10.79 ± 2.66

**FIGURE 4:**
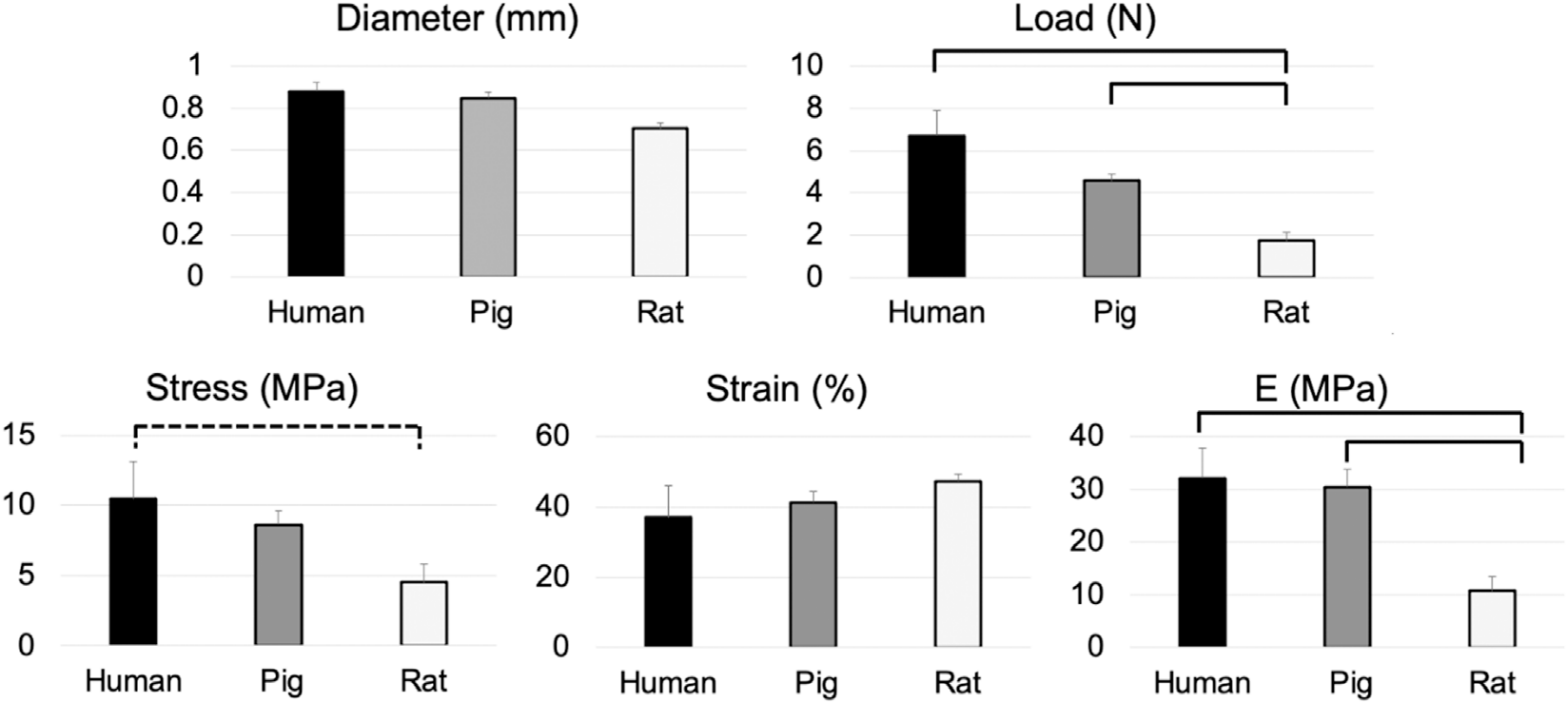
Average + SEM values of the Diameter (mm), Failure load (N), Failure stress (MPa), Strain at failure (%), and Modulus of Elasticity (E, MPa) reported in the age-equivalent human, pig, and rat sural nerves. Solid brackets above the two groups indicate significant differences (*p* < 0.05) between the groups. The dotted bracket above the two groups indicates moderate significance (*p* < 0.1) between the two groups.

The sural nerve diameters measured for human and pig samples were similar and were larger than the rat sural nerve diameters (Figure 4). However, there were no significant differences in the nerve diameters between the three species. When comparing the biomechanical tensile properties of the samples, the observed failure load and calculated failure stress, corresponding strain, and E values were highest in humans, followed by pig and then rat nerves. These differences were significant for failure load (*p* < 0.01) when comparing the rat sural nerve to the human and pig nerves. No differences were observed between human and pig sural nerves. The reported failure stress was also highest in the human nerve, followed by pig and then rat sural nerve. A moderate significant difference (*p* = 0.07) was observed between the human and rat sural nerves. No significant differences in the failure stresses were observed between the human and pig nerves, and pig, and rat nerves. For the strain values observed at failure stress, no significant differences were observed between the human, pig, and rat sural nerves. Finally, the E (modulus of elasticity) values were also significantly higher in the human and pig sural nerves when compared to rat nerve with no significant difference observed between the human and pig sural nerves (Figure 4).

### 3.2 Structural damage

**FIGURE 5:**
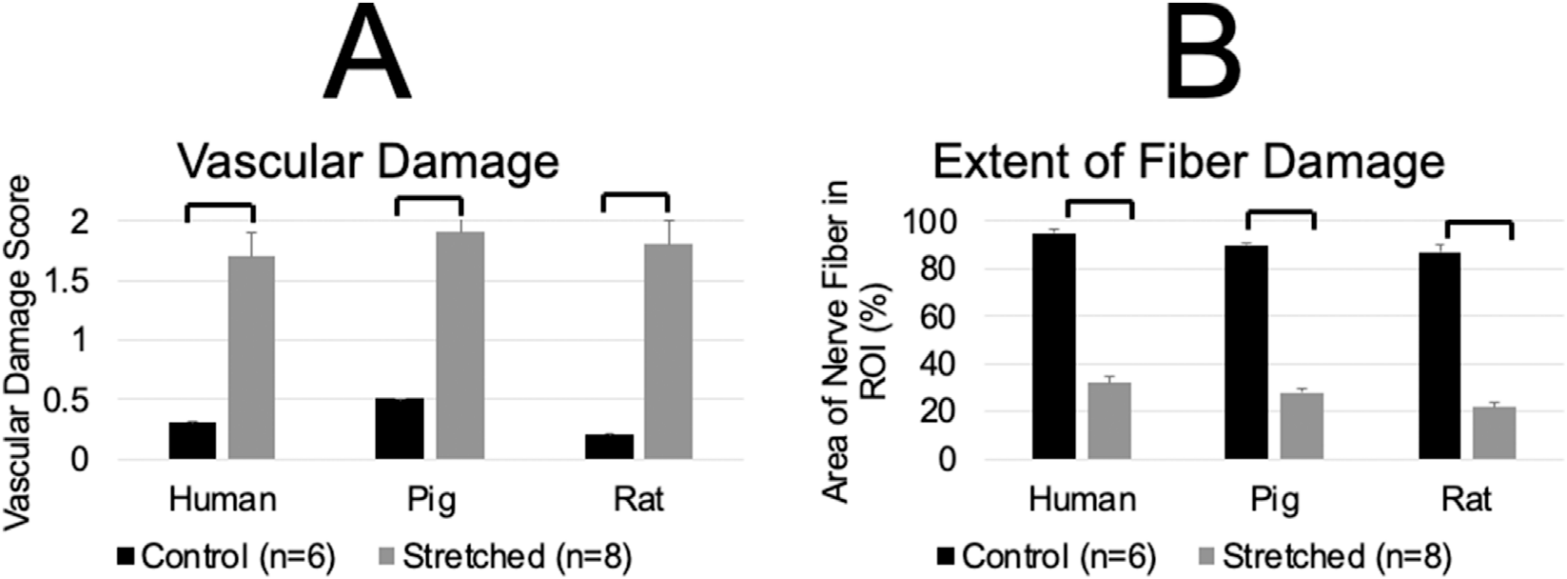
Histological data summary for control (n = 6) and stretched (n = 8) sural nerves of age-equivalent human, pig, and rat species. **(A)** Extent of vascular damage. Score 0- No Damage, 1-Moderate Damage and 2-Severe Damage. **(B)** Extent of fiber damage by measuring the extent of fiber in the region of interest (ROI). Increased spacing and broken/defragmented fiber led to a decrease in the % of nerve fiber in the ROI when compared to the control. A significant difference was observed between the control and stretched nerves. No species-specific significant differences were observed within the control or stretched groups. Solid brackets above the two groups indicate significant differences (*p* < 0.05) between the groups.

When comparing the stretched versus the control nerve tissue, blood vessel rupture, and fiber damage were evident along the entire length of the stretched nerve in all three species (Figure 5). The blood vessels of the stretched nerves had broken walls with scattered RBCs within the tissue. The fibers of the stretched nerve had lost their wavy behavior, demonstrated increased spacing, and were mostly defragmented in several regions along the length of the nerve. No species-specific significant differences were observed in any studied parameters of the stretched sural nerve. Also, for each studied species, the vascular damage scores and the extent of fiber damage were significantly higher in the stretched nerve than in their control nerve (*p* < 0.01).

## 4 Discussion

The peripheral nerve is a complex structure consisting of bundles of fascicles that are surrounded by load-bearing coverings, the epineurium, and the perineurium. The biomechanical responses of the nerve tissue can vary depending upon the number and arrangement of its fascicles (Sunderland and Bradley, 1961), the structural elements of its extracellular matrix such as collagen and elastin (Ushiki and Ide, 1990; Tasslwe et al., 1994) as well as the integrity of its coverings (Walbeehm et al., 2004). Furthermore, the internal fluid pressure maintained by the impermeable perineurium (Low et al., 1977) of the nerve plays a critical role in its viscoelastic behavior. Despite the possibility of high variability within the peripheral nerve of various species (Martinez-Pereira and Zancan, 2015), translational research heavily relies on experimental animal models such that 91% of reported PNI studies utilize rodents, and the remaining 9% use large animal models (Lopes et al., 2023). Experimental animal models for PNI serve as good surrogates to help understand the pathophysiology as well as to aid in investigating translational repair strategies. Acceleration in translational research efforts heavily relies on the validation of these commonly used animal models and identifying those that most closely resemble human tissue behavior. Limited information is available on how the biomechanical responses of commonly used small animal models such as rats/mice and less commonly used large animal models such as pigs/dogs/cats compare to that of the human peripheral nerve. This study aimed to fill this critical gap by comparing the biomechanical responses of human sural nerve to an age-equivalent pig (large animal model) and rat (small animal model) sural nerve.

In the current study, the biomechanical responses reported similar trends in stress-strain behavior of the human, pig, and rat sural nerves. Tensile stretching reported a linear increase in stress with increasing strain when subjected to a stretch rate of 500 mm/min, until a complete failure, which is evident by an abrupt drop in stress (Figure 3, rat and pig nerve shows an example of such response). In some cases, a partial tear followed by complete failure was also observed as evident from a two-peak response in the stress-strain plot (Figure 3, human nerve shows an example of such response). While the stress-strain responses were similar in their trends, the rat sural nerve did report significantly lower failure properties (i.e., failure load) and a lower modulus of elasticity (E, measure of elasticity) when compared to human and pig sural nerves. These reported differences can be attributed to both the structural differences as well as the *in-situ* length of the tissue. Species-specific differences in E values have been reported previously such that rabbit nerves have been reported to be more extensible than those from pigs (Koike, 1987). The lower E values in rat nerve confirms the higher stretchability of the rat tissue when compared to human and pig nerve tissue. The higher extensibility of rat peripheral nerves compared to other species likely arises from differences in extracellular matrix composition and microstructural organization. Rats may have lower collagen content or a higher proportion of more compliant collagen types (e.g., collagen III), reducing stiffness. We also observed higher strains at failure stress in the rat nerve when compared to human and pig nerves, although not statistically significant. Overall, these findings confirm the species-specific difference in the biomechanical responses of the rat and pig as well as human peripheral nerve tissue. Furthermore, no differences in the biomechanical responses of the age-equivalent human and pig tissue behavior support the use of the pig as a promising surrogate animal model for PNI translational research.

When comparing species-specific structural changes within the stretched peripheral nerve tissue, all stretched nerves reported a loss of their wavy morphology. Increased spacing and fragmented fibers were also observed along the length of all the stretched nerves. Observed vascular damage including broken blood vessels and scattered red blood cells was observed in the stretched nerves of all three species. These findings confirm that at the macroscopic level, the structural responses to stretch are similar within the peripheral nerves of various species. Previous studies have reported fading of the spiral bands of Fontana with stretching (Clarke and Bearn, 1972; Williams and Hall, 1970; Pourmand et al., 1994), the incisures of Schmidt-Lantermann as sites at which the myelin sheath telescopes to accommodate stretching (Glees, 1943), and changes in the covering of the load-bearing nerve structures when subjected to mechanical forces (Tasslwe et al., 1994). Since, weaker mechanical responses were reported in the rat peripheral nerve, despite any differences in the macrolevel structural damage, future in-depth histological and histochemical studies are needed that further outline the underlying mechanism of nerve injury response when stretched to non-physiological limits in various PNI animal models.

In summary, this study is the first to report and compare the biomechanical responses in fresh sural nerves of human infants, and age-equivalent pig and rat animal models. Cross-species comparison of viscoelastic behavior refines the mechanical fidelity of animal models, enhances translation of experimental findings, and informs clinical design standards for nerve repair and protective strategies. In the current study, no significant differences in the failure properties of the age-equivalent human and pig sural nerves were observed. However, rat sural nerves reported significantly weaker biomechanical properties. The observed structural damage was similar in the three studied species. A serious limitation of the current study is the ex-vivo biomechanical testing environment, which might alter the physiological biomechanical behavior of the studied nerves. Another limitation is the lack of information on the micromechanical behavior of fascicles, axons, and extracellular matrix since whole-nerve mechanics was studied.

In recent years, there has been a considerable scale-up of *in vivo* testing in large animal models due to their anatomical and physiological similarities to humans, however anatomical location of the studied nerve should also be considered (Lopes et al., 2023). The primary goal of translational relevance of animal models commonly used in nerve injury and repair research can only be achieved by critically selecting the appropriate animal model that accounts for existing variabilities. This study offers biomechanical data to strongly support these efforts and validates the use of the pig animal model as a promising surrogate to advance translational PNI research.

## 5 Conclusion

This section is not mandatory but can be added to the manuscript if the discussion is unusually long or complex.

## Data Availability

The original contributions presented in the study are included in the article/supplementary material, further inquiries can be directed to the corresponding author.

## References

[B1] Birch R. Achan P. (2000). Peripheral nerve repairs and their results in children. Hand Clin. 16 (4), 579–595. 10.1016/S0749-0712(21)00219-5 11117049

[B2] Clarke E. Bearn J. G. (1972). The spiral nerve bands of Fontana. Brain 95 (1), 1–20. 10.1093/brain/95.1.1 4554004

[B3] Destandau J. Micallef J. P. Rabischong P. (1986). An experimental study of traction on the cervical spinal nerves. Surg. Radiol. Anat. 8 (3), 197–204. 10.1007/BF02427849 3099411

[B4] D’Andrea C. R. Alfraihat A. Singh A. Anari J. B. Cahill P. J. Schaer T. (2021). Part 1. Review and meta‐analysis of studies on modulation of longitudinal bone growth and growth plate activity: a macro‐scale perspective. J. Orthop. Res. 39 (5), 907–918. 10.1002/jor.24976 33377536

[B5] Fung Y. C. (1993). Biomechanics: mechanical properties of living tissues. New York, NY: Springer New York. 10.1007/978-1-4757-2257-4

[B6] Giannessi E. Stornelli M. R. Sergi P. N. (2017). A unified approach to model peripheral nerves across different animal species. PeerJ 5, e4005. 10.7717/peerj.4005 29142788 PMC5683050

[B7] Glees P. (1943). Observations on the structure of the connective tissue sheaths of cutaneous nerves. J. Anat. 77 (Pt 2), 153–159. Available online at: http://www.ncbi.nlm.nih.gov/pubmed/17104922. 17104922 PMC1252752

[B8] Gör L. Rydevik B. (1973). Effects of stretching the tibial nerve of the rabbit. J. Bone Jt. Surg. Br. 55 (2), 390–401. 10.1302/0301-620X.55B2.390 4707307

[B9] Kallakuri S. Singh A. Chen C. Cavanaugh J. M. (2004). Demonstration of substance P, calcitonin gene-related peptide, and protein gene product 9.5 containing nerve fibers in human cervical facet joint capsules. Spine (Phila Pa 1976) 29 (11), 1182–1186. 10.1097/00007632-200406010-00005 15167655

[B10] Kawai H. Ohta I. Masatomi T. Kawabata H. Masada K. Ono K. (1989). Stretching of the brachial plexus in rabbits. Acta Orthop. Scand. 60 (6), 635–638. 10.3109/17453678909149592 2624080

[B11] Kleinrensink G. J. Stoeckart R. Mulder P. G. Hoek G. Broek T. Vleeming A. (2000). Upper limb tension tests as tools in the diagnosis of nerve and plexus lesions. Anatomical and biomechanical aspects. Clin. Biomech. (Bristol) 15 (1), 9–14. 10.1016/s0268-0033(99)00042-x 10590339

[B12] Koike H. (1987). The extensibility of aplysia nerve and the determination of true axon length. J. Physiol. 390 (1), 469–487. 10.1113/jphysiol.1987.sp016712 3502142 PMC1192192

[B13] Kwan M. K. Wall E. J. Massie J. Garfin S. R. (1992). Strain, stress and stretch of peripheral nerve rabbit experiments *in vitro* and *in vivo* . Acta Orthop. Scand. 63 (3), 267–272. 10.3109/17453679209154780 1609588

[B14] Lopes B. Coelho A. Alvites R. Sousa A. C. Sousa P. Moreira A. (2023). Animal models in peripheral nerve transection studies: a systematic review on study design and outcomes assessment. Regen. Med. 19, 189–203. 10.2217/rme-2023-0102 37855207 PMC12997969

[B15] Low P. Marchand G. Knox F. Dyck P. J. (1977). Measurement of endoneurial fluid pressure with polyethylene matrix capsules. Brain Res. 122 (2), 373–377. 10.1016/0006-8993(77)90305-5 837238

[B16] Millesi H. Zöch G. Reihsner R. (1995). Mechanical properties of peripheral nerves. Clin. Orthop. Relat. Res. 314, 76–83. 10.1097/00003086-199505000-00011 7634654

[B17] Ma Z. Hu S. Tan J. S. Myer C. Njus N. M. Xia Z. (2013). *In vitro* and *in vivo* mechanical properties of human ulnar and median nerves. J. Biomed. Mater Res. Part A 101A (9), 2718–2725. 10.1002/jbm.a.34573 23568572

[B18] Marani E. van Leeuwen J. L. Spoor C. W. (1993). The tensile testing machine applied in the study of human nerve rupture: a preliminary study. Clin. Neurol. Neurosurg. 95, 33–35. 10.1016/0303-8467(93)90032-C 8467594

[B19] Martinez-Pereira M. A. Zancan D. M. (2015). “Comparative anatomy of the peripheral nerves,” in Nerves and nerve injuries (Elsevier), 55–77.

[B20] Narakas A. O. (1993). Lesions found when operating traction injuries of the brachial plexus. Clin. Neurol. Neurosurg. 95, S56–S64. 10.1016/0303-8467(93)90037-h 8467598

[B21] Ochs S. Pourmand R. Si K. Friedman R. N. (2000). Stretch of Mammalian nerve *in vitro:* effect on compound action potentials. J. Peripher Nerv. Syst. 5 (4), 227–235. 10.1046/j.1529-8027.2000.00025.x 11151983

[B22] Orozco V. Balasubramanian S. Singh A. (2024). Direct linear transformation for the measurement of *in-situ* peripheral nerve strain during stretching. J. Vis. Exp. 203. 10.3791/65924 38284518

[B23] Pourmand R. Ochs S. Jersild R. A. (1994). The relation of the beading of myelinated nerve fibers to the bands of fontana. Neuroscience 61 (2), 373–380. 10.1016/0306-4522(94)90238-0 7969916

[B24] Rydevik B. L. Kwan M. K. Myers R. R. Brown R. A. Triggs K. J. Woo S. L. (1990). An *in vitro* mechanical and histological study of acute stretching on rabbit tibial nerve. J. Orthop. Res. 8 (5), 694–701. 10.1002/jor.1100080511 2388109

[B25] Singh A. (2017). Extent of impaired axoplasmic transport and neurofilament compaction in traumatically injured axon at various strains and strain rates. Brain Inj. 31 (10), 1387–1395. 10.1080/02699052.2017.1321781 28650256

[B26] Singh A. Lu Y. Chen C. Kallakuri S. Cavanaugh J. M. (2006a). A new model of traumatic axonal injury to determine the effects of strain and displacement rates. Stapp Car Crash J. 50, 601–623. 10.4271/2006-22-0023 17311179

[B27] Singh A. Lu Y. Chen C. Cavanaugh J. M. (2006b). Mechanical properties of spinal nerve roots subjected to tension at different strain rates. J. Biomech. 39 (9), 1669–1676. 10.1016/j.jbiomech.2005.04.023 15996674

[B28] Singh A. Kallakuri S. Chen C. Cavanaugh J. M. (2009). Structural and functional changes in nerve roots due to tension at various strains and strain rates: an *in-vivo* study. J. Neurotrauma 26 (4), 627–640. 10.1089/neu.2008.0621 19271962

[B29] Singh A. Shaji S. Delivoria-Papadopoulos M. Balasubramanian S. (2018). Biomechanical responses of neonatal brachial plexus to mechanical stretch. J. Brachial Plex. Peripher Nerve Inj. 13 (01), e8–e14. 10.1055/s-0038-1669405 30210576 PMC6133693

[B30] Sunderland S. (1951). A classification of peripheral nerve injuries producing loss of function. Brain 74 (4), 491–516. 10.1093/brain/74.4.491 14895767

[B31] Sunderland S. Bradley K. C. (1961). Stress-strain phenomena in human spinal nerve roots. Brain 84 (1), 120–124. 10.1093/brain/84.1.120

[B32] Takai S. Dohno H. Watanabe Y. Yoshino N. Ogura T. Hirasawa Y. (2002). *In situ* strain and stress of nerve conduction blocking in the brachial plexus. J. Orthop. Res. 20 (6), 1311–1314. 10.1016/s0736-0266(02)00080-3 12472245

[B33] Tasslwe P. L. Dellon A. L. Canoun C. (1994). Identification of elastic fibres in the peripheral nerve. J. Hand Surg. Am. 19 (1), 48–54. 10.1016/0266-7681(94)90049-3 8169479

[B34] Topp K. S. Boyd B. S. (2006). Structure and biomechanics of peripheral nerves: nerve responses to physical stresses and implications for physical therapist practice. Phys. Ther. 86 (1), 92–109. 10.1093/ptj/86.1.92 16386065

[B35] Ushiki T. Ide C. (1990). Three-dimensional organization of the collagen fibrils in the rat sciatic nerve as revealed by transmission- and scanning electron microscopy. Cell Tissue Res. 260 (1), 175–184. 10.1007/BF00297503 2340581

[B36] Walbeehm E. T. Afoke A. de Wit T. Holman F. Hovius S. E. R. Brown R. A. (2004). Mechanical functioning of peripheral nerves: linkage with the “mushrooming” effect. Cell Tissue Res. 316 (1), 115–121. 10.1007/s00441-004-0867-9 14986104

[B37] Wall E. J. Massie J. B. Kwan M. K. Rydevik B. L. Myers R. R. Garfin S. R. (1992). Experimental stretch neuropathy. Changes in nerve conduction under tension. J. Bone Jt. Surg. Br. 74 (1), 126–129. 10.1302/0301-620X.74B1.1732240 1732240

[B38] Williams P. L. Hall S. M. (1970). *In vivo* observations on mature myelinated nerve fibres of the mouse. J. Anat. 107 (Pt 1), 31–38. Available online at: http://www.ncbi.nlm.nih.gov/pubmed/5473291. 5473291 PMC1234162

[B39] Zapałowicz K. Radek A. (2000). Mechanical properties of the human brachial plexus. Neurol. Neurochir. Pol. 34 (6), 89–93. Available online at: http://www.ncbi.nlm.nih.gov/pubmed/11452861. 11452861

[B40] Zapałowicz K. Radek A. (2005). Experimental investigations of traction injury of the brachial plexus. Model and results. Ann. Acad. Med. Stetin. 51 (2), 11–14. Available online at: http://www.ncbi.nlm.nih.gov/pubmed/16519090. 16519090

